# Nationwide database study of postoperative sequelae and in-hospital mortality in super-elderly hip fracture patients

**DOI:** 10.1007/s00774-024-01564-w

**Published:** 2024-11-07

**Authors:** Yu Mori, Kunio Tarasawa, Hidetatsu Tanaka, Naoko Mori, Kiyohide Fushimi, Toshimi Aizawa, Kenji Fujimori

**Affiliations:** 1 Department of Orthopaedic Surgery, Tohoku University Graduate School of Medicine, 1-1 Seiryo-Machi, Aoba-ku, Sendai, Miyagi 980-8574 Japan; 2 Department of Health Administration and Policy, Tohoku University Graduate School of Medicine, 2-1 Seiryo-Machi, Aoba-ku, Sendai, Miyagi 980-8574 Japan; 3 Department of Radiology, Akita University Graduate School of Medicine, 1-1-1 Hondo, Akita, Akita, 010-8543 Japan; 4 Department of Health Policy and Informatics, Tokyo Medical and Dental University Graduate School of Medicine and Dental Sciences, 1-5-45 Yushima, Bunkyo-ku, Tokyo, 113-8519 Japan

**Keywords:** Hip fracture, Mortality, Nationwide database, Osteoporosis, Super-elderly

## Abstract

**Introduction:**

The risk of postoperative sequelae and in-hospital mortality in Japanese patients aged 90 years and older with hip fractures is unexplored. This study aims to use a comprehensive medical claims database in Japan to compare super-elderly patients aged 90 years and older with elderly aged 65–89 and clarify the risk of sequelae and in-hospital mortality in super-elderly patients.

**Materials and methods:**

We retrospectively analyzed the Diagnosis Procedure Combination (DPC) database for all of Japan from April 2016 to March 2022. Medical records from approximately 1100 DPC-related hospitals were provided with consistent consent during this period. In this study, we focused on super-elderly patients and examined the association with the risk of postoperative pneumonia, pulmonary embolism, myocardial infarction, urinary tract infection, acute renal dysfunction, subsequent cognitive dysfunction, and in-hospital mortality after one-to-one propensity score matching.

**Results:**

After performing propensity score matching based on sex and comorbidities, 129,953 pairs of patients were identified. These pairs were compared to elderly and super-elderly patients. The results of this study showed that compared with hip fractures in the elderly, hip fractures in the super-elderly were associated with an increased risk of pneumonia, urinary tract infection, acute renal dysfunction, subsequent cognitive dysfunction, and in-hospital mortality after adjustment for confounders. The odds ratio of in-hospital mortality was 2.190 (95% CI 2.062–2.325).

**Conclusion:**

As it has been shown that super-elderly patients with hip fractures are at greater risk of respiratory and urinary tract infections and increased in-hospital mortality, careful attention should be required for perioperative management.

**Supplementary Information:**

The online version contains supplementary material available at 10.1007/s00774-024-01564-w.

## Introduction

Hip fractures are a common orthopedic injury in the elderly, with high morbidity and mortality rates [1, 2]. The number of hip fractures worldwide is expected to reach 4,500,000 by 2050 [3]. The elderly population in the United States continues to grow, and the incidence of proximal femur fractures is expected to double from 250,000 in 1990 to 500,000 in 2040 [4]. In Japan, where the population is aging, there are an estimated 13 million patients with osteoporosis [5], and the number of patients with proximal femur fractures is estimated to be 250,000 [6]. Therefore, proximal femur fractures due to osteoporosis are a global problem regardless of country or region. More than 90% of these fractures occur in patients aged 65 years or older. In patients with a hip fracture, comorbidity also increases over time. The 30-day mortality rate for hip fractures is estimated to be 4.0–5.4% [7], and the 1-year mortality rate is estimated to be 25% [8].

Due to the aging population, the number of hip fracture surgeries in the super-elderly population aged 90 years and older is also increasing, and the treatment outcomes of hip fracture cases in the super-elderly population have been reported in relatively small studies [9–15]. The 30-day mortality rate for the surgery group has been reported to range from 1.7% to 9.6%, and it has been reported that the mortality rate for the surgery group is significantly lower than that for the non-surgery group [15]. However, although it has been reported that the perioperative risk of hip fracture surgery is higher in super-elderly patients than in younger patients [9], there have not yet been sufficient detailed studies of postoperative complications of hip fractures in super-elderly patients.

A study using the Diagnosis Procedure Combination (DPC) database of Japanese patients with hip fractures reported the effects of performing surgery on the day of admission or the following day in reducing the incidence of complications, as well as comparing bipolar hemiarthroplasty surgery with total hip arthroplasty surgery for femoral neck fractures [16–18]. On the other hand, the risk of complications in super-elderly patients with hip fractures has not yet been investigated. Therefore, in this study, we will use a large database of Japanese hip fracture cases to examine the incidence of postoperative complications and mortality during hospitalization in super-elderly patients aged 90 years and older compared with those aged 65 to 89 years.

## Materials and methods

### Study design

This retrospective study was conducted in accordance with the ethical standards of the Declaration of Helsinki and obtained ethical approval from both Tokyo Medical and Dental University (approval number: M2000-788) and Tohoku University (approval number: 2021–1-1082). Data were retrospectively collected from the Japanese National Administrative DPC reimbursement system database [19]. At the time of admission, each hospital obtained comprehensive informed consent from the patients, which included authorizations for the proposed treatment methods and academic use of the data collected during their treatments. In addition, the manuscript contains no identifiable information about the participants. The study period was from April 2016 to March 2022. This study is a comprehensive survey of DPC-participating hospitals throughout Japan. During this period, approximately 1100 hospitals covered by the DPC system regularly submitted medical records and consented to their use for research purposes. Patients who underwent surgical treatment for hip fractures at these hospitals across Japan were included in the analysis. This data represents the actual treatment situation for hip fractures in Japan. This clinical study focused on hip fractures in elderly people aged 65 years and over, and in particular, it focused on the incidence of postoperative sequelae and short-term mortality in the super-elderly with hip fractures. Postoperative sequelae were evaluated for pneumonia, pulmonary embolism, myocardial infarction, acute renal dysfunction, urinary tract infection, and postoperative cognitive dysfunction. Postoperative cognitive dysfunction was extracted using the International Statistical Classification of Diseases, Tenth Revision (ICD-10) codes F010, F011, F012, F019, F03, F107, G238, G300, G301, G308, G309, G310, and G318 for cognitive dysfunction and delirium that occurred during the postoperative period. Hip fractures were classified according to ICD-10 codes: S7200 for femoral neck fractures, S7210 for trochanteric fractures, and S7220 for subtrochanteric fractures. Patients included in the hip fracture cohorts were selected from a registry that organized entries based on three criteria: [1] principal diagnosis, [2] primary reason for admission, and [3] condition that utilized the most medical resources. The exclusion criteria are patients under 65 years of age and patients with hip fractures treated conservatively.

### Propensity score matching

We defined those aged 65 years and older as elderly and those aged 90 years and older as super-elderly. In this study, we compared hip fracture patients aged 65 to 89 years with those aged 90 years and older. A one-to-one propensity score (PS) matching was performed between hip fracture patients aged 65 to 89 years and those aged 90 years and older. Confounders adjusted for in the analysis were sex, the Charlson comorbidity index, and comorbid conditions, including hypertension, dementia, ischemic heart disease, cerebrovascular disease, chronic renal dysfunction, chronic lung disease, and diabetes. The discriminative ability of the model was assessed using C-statistics. PS estimates facilitated nearest-neighbor matching without replacement, using these scores as calipers set at 0.2 times the standard deviation of the PS estimate [20]. This approach generated matched pairs that formed elderly and super-elderly groups based on PS matching.

### Statistical analyses

Data are presented as mean ± standard deviation. Differences between the two groups were analyzed using the χ2 test and Student's t-test for each clinical parameter, comparing elderly and super-elderly patients. We performed a multivariate logistic regression analysis of variables associated with mortality during hospitalization and examined the association with super-elderly age (90 years or older), sex, surgery within two days of hospitalization, and comorbidities and identified independent risk factors. The log-rank test was used to compare the difference in survival between groups. Due to the large sample size of the study, strict significance thresholds were set. All statistical tests were two-tailed, and p-values < 0.001 were considered statistically significant. Statistical analyses were performed using JMP version 17 (SAS, Cary, NC, USA).

## Results

**Fig. 1:**
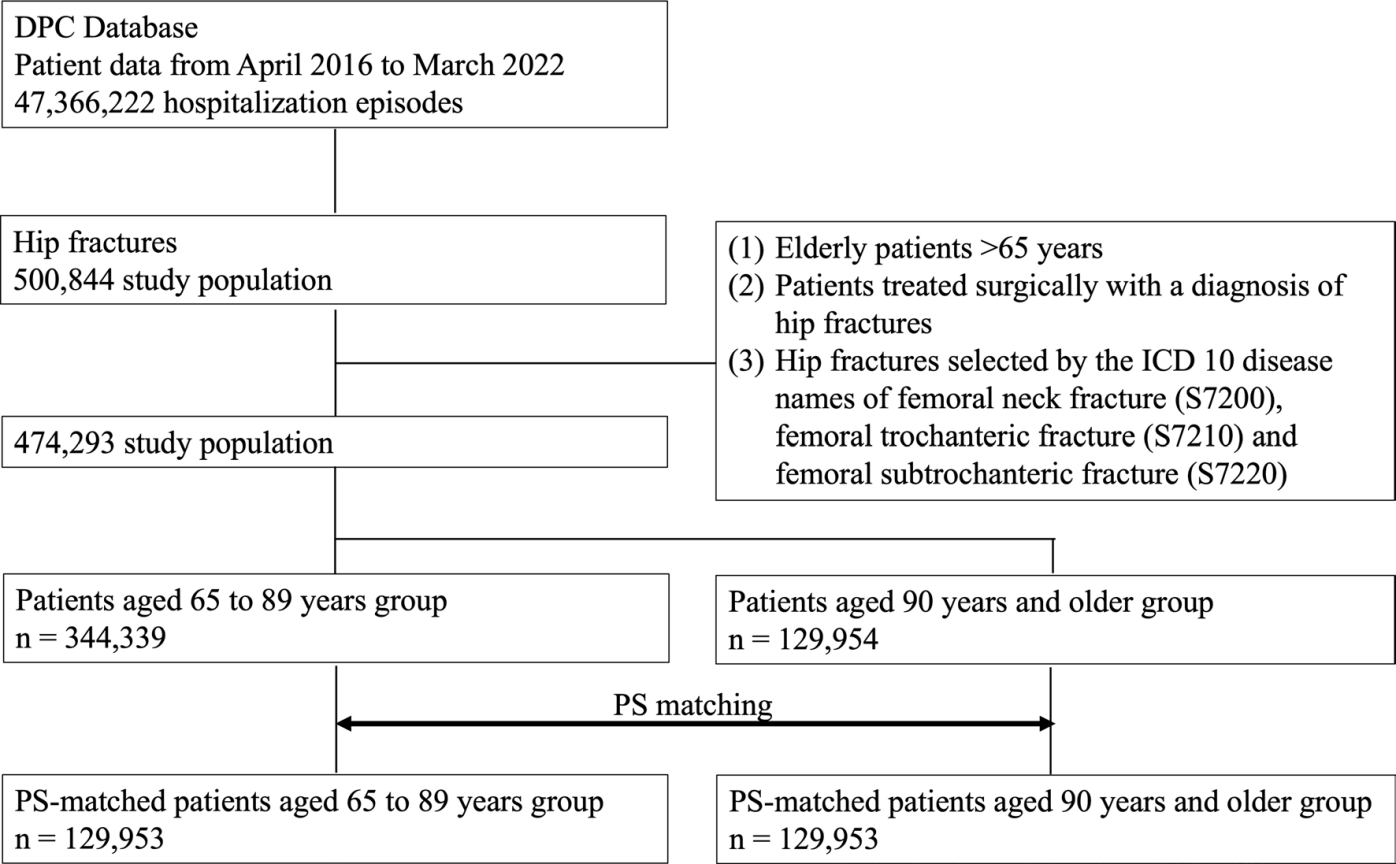
Flow diagram of patient selection for super-elderly patients with hip fracture and propensity score (PS) matching. This diagram shows the method for extracting target patients from the DPC database and PS matching for patients aged 65 to 89 with hip fractures and patients aged 90 and over with hip fractures

Figure 1 presents a schematic model of the patient selection process: A total of 500,844 patients who met the inclusion and exclusion criteria were selected from the patient data from April 2016 to March 2022. Of these, 344,339 were patients aged 65 to 89 years, and 129,954 were patients aged 90 years and older. There were 129,953 cases in each of the elderly and super-elderly groups after one-to-one propensity score matching based on sex, the Charlson comorbidity index, and comorbidity.

Table 1 shows the characteristics of elderly and super-elderly patients with hip fractures. Before PS matching, significant differences were observed between the two groups in terms of sex and comorbidities. In the super-elderly group, there was a higher prevalence of comorbidity of hypertension, dementia, and ischemic heart disease. In addition, the body mass index was lower in the super-elderly patients' group. In the super-elderly group, the most common type of fracture was a femoral trochanteric fracture, while in the elderly group, the most common type of fracture was a femoral neck fracture. In relation to the type of fracture, the super-elderly group had more cases of osteosynthesis surgery, while the elderly group had more cases of bipolar hemiarthroplasty. The rate of early surgery within two days of hospitalization was high in the super-elderly group, and the length of stay tended to be short. On the other hand, the super-elderly group used more blood transfusions. The C statistic was calculated to be 0.713. After PS matching, the standardized mean differences for all measured parameters were less than 0.1, indicating well-balanced groups. After one-to-one PS matching, there was no difference between the two groups in the parameters of sex and comorbidities. The results for the comparisons of body mass index, fracture type, type of surgery performed, rate of early surgery, amount of blood transfusion, and length of stay were the same as before PS matching.

**Table 1:** Characteristics of patients before and after propensity score matching

	Before PS matching			After PS matching			*p*-value
	Elderly	Super-elderly	*p*-value	Elderly	Super-elderly	SMD	
n	344,339	129,954		129,953	129,953		
Sex							
Men	84,646 (24.6%)	18,518 (14.3%)	< 0.0001	18,565 (14.3%)	18,518 (14.3%)	0.0011	0.79
Women	259,693 (75.4%)	111,436 (85.7%)		111,388 (85.7%)	111,435 (85.7%)		
Comorbidities							
Charlson comorbidity index	1.2 ± 1.3	1.2 ± 1.3	0.46	1.2 ± 1.3	1.2 ± 1.3	0.0006	0.89
Hypertension	127,233 (37.0%)	55,579 (42.8%)	< 0.0001	55,583 (42.8%)	55,578 (42.8%)	0.0002	0.98
Dementia	68,095 (19.8%)	36,126 (27.8%)	< 0.0001	36,108 (27.8%)	36,125 (27.8%)	0.0003	0.94
Diabetes	71,424 (20.7%)	16,443 (12.7%)	< 0.0001	16,485 (12.7%)	16,443 (12.7%)	0.0009	0.81
Cerebrovascular disease	36,193 (10.5%)	12,539 (9.6%)	< 0.0001	12,526 (9.6%)	12,539 (9.6%)	0.0003	0.93
Ischemic heart disease	26,294 (7.6%)	11,872 (9.1%)	< 0.0001	11,847 (9.1%)	11,871 (9.1%)	0.0006	0.87
Chronic renal dysfunction	19,550 (5.7%)	6013 (4.6%)	< 0.0001	6019 (4.6%)	6012 (4.6%)	0.0003	0.95
Chronic lung disease	5623 (1.6%)	1547 (1.2%)	< 0.0001	1515 (1.2%)	1547 (1.2%)	0.0022	0.56

						χ2 statics	*p*-value
Age	81.1 ± 6.2	93.2 ± 2.8	< 0.0001	81.6 ± 6.0	93.2 ± 2.8		< 0.0001
Body mass index	20.7 ± 5.0	20.0 ± 5.4	< 0.0001	20.6 ± 4.2	20.0 ± 5.4		< 0.0001
Fracture type							
Femoral neck	190,868 (55.4%)	47,557 (36.6%)	< 0.0001	71,513 (55.0%)	47,557 (36.6%)	9082	< 0.0001
Femoral trochanteric	146,098 (42.4%)	79,542 (61.2%)		55,693 (42.9%)	79,541 (61.2%)		
Femoral subtrochanteric	7373 (2.2%)	2855 (2.2%)		2747 (2.1%)	2855 (2.2%)		
General anesthesia	213,311 (62.0%)	75,104 (57.8%)	< 0.0001	80,553 (62.0%)	75,103 (57.8%)	475.9	< 0.0001
Surgery							
ORIF	202,928 (58.9%)	95,522 (73.5%)	< 0.0001	76,919 (59.2%)	95,521 (73.5%)	3758	< 0.0001
BHA	135,366 (39.3%)	34,063 (26.2%)		50,875 (39.1%)	34,063 (26.2%)		
THA	6045 (1.8%)	369 (0.3%)		2159 (1.7%)	369 (0.3%)		
Surgery within 2 days of admission	160,591 (46.6%)	66,345 (51.1%)	< 0.0001	61,179 (47.1%)	66,345 (51.1%)	411.5	< 0.0001
Surgery after 2 days of admission	183,748 (53.4%)	63,609 (48.9%)		68,774 (52.9%)	63,608 (48.9%)		
Blood transfusion (Units)	0.39 ± 1.02	0.60 ± 1.17	< 0.0001	0.40 ± 1.03	0.60 ± 1.17		< 0.0001
Length of Hospitalization (Days)	35.6 ± 28.6	34.9 ± 26.6	< 0.0001	35.4 ± 28.0	34.9 ± 26.6		< 0.0001

One-to-one PS matching was performed

Data is shown as mean ± standard deviation; P-values of < 0.001 are considered significant by the Student’s-t test and χ2 test; BHA means bipolar hemiarthroplasty; ORIF means open reduction and internal fixation; PS means propensity score: SMD means standard mean difference; THA means total hip arthroplasty

Table 2 shows the use of antithrombotic drugs and osteoporosis treatments. The use of antithrombotic drugs was 49.2% in both groups, and there was no significant difference in the use of each drug between the two groups. Active forms of vitamin D and weekly bisphosphonates were the most common treatments for osteoporosis. Interestingly, eldecalcitol was used more frequently in the elderly group and alfacalcidol more frequently in the super-elderly group.

**Table 2:** Anticoagulants and osteoporosis medications

	Elderly	Super-elderly	χ2 statics	*p*-value
Antithrombotic drugs				
All anticoagulants	64,002 (49.2%)	63,985 (49.2%)	0.004	0.94
Edoxaban tosilate hydrate	34,284 (26.4%)	34,421 (26.5%)	0.37	0.54
Fondaparinux sodium	1801 (1.4%)	1660 (1.3%)	5.8	0.016
Enoxaparin sodium	4883 (3.8%)	4851 (3.7%)	0.1	0.74
Aspirin	15,667 (12.1%)	15,626 (12.0%)	0.06	0.8
Warfarin potassium	5016 (3.9%)	4957 (3.8%)	0.36	0.55
Other anticoagulants	10,767 (8.3%)	10,766 (8.3%)	0.0001	0.99
Osteoporosis treatment				
Daily bisphosphonates	306 (0.2%)	273 (0.2%)	1.9	0.17
Weekly bisphosphonates	10,179 (7.8%)	8237 (6.3%)	220.8	< 0.0001
Monthly bisphosphonates (oral)	3663 (2.8%)	3010 (2.3%)	65.7	< 0.0001
Monthly bisphosphonates (iv)	916 (0.7%)	929 (0.7%)	0.09	0.76
Yearly bisphosphonates (iv)	209 (0.2%)	200 (0.2%)	0.2	0.66
Daily teriparatide	1502 (1.2%)	1244 (1.0%)	24.5	< 0.0001
Weekly teriparatide	834 (0.6%)	749 (0.6%)	4.6	0.03
Biweekly teriparatide	225 (0.2%)	168 (0.1%)	8.3	0.004
Denosumab	488 (0.4%)	463 (0.4%)	0.66	0.42
Eldecalcitol	12,973 (10.0%)	11,115 (8.6%)	158.1	< 0.0001
Alfacalcidol	13,903 (10.7%)	14,547 (11.2%)	16.4	< 0.0001
SERM	2082 (1.6%)	1979 (1.5%)	2.7	0.1

*P*-values of < 0.001 are considered significant by the χ2 test; iv means intravenous injection; SERM means selective estrogen receptor modulator

Table 3 shows the results of the analysis of the risk of sequelae in the super-elderly group compared to the elderly group. The risks of pneumonia, acute renal dysfunction, urinary tract infection, subsequent cognitive dysfunction, and mortality during hospitalization were significantly higher in the super-elderly group, with odds ratios of 1.636 (95% CI 1.565–1.709), 1.683 (95% CI 1.399–2.026), 1.378 (95% CI 1.321–1.438), 1.443 (95% CI 1.354–1.538), and 2.190 (95% CI 2.062–2.325), respectively. On the other hand, the odds ratio for pulmonary embolism was significantly lower in the super-elderly group. In the appendix, we have attached a table summarizing the number of occurrences of each sequela and postoperative mortality in three age groups: 65 to 79 years old, 80 to 89 years old, and 90 years old and over (Supplementary Table 1).

**Table 3:** Risk assessment of postoperative sequelae and in-hospital mortality in super-elderly patients with hip fractures

Complication	Total	Super-elderly	Odds Ratio (95% CI)	χ2 statics	*p*-value
Pneumonia	8684	5356 (61.7)	1.636 (1.565–1.709)	494.4	< 0.0001
Pulmonary embolism	11,398	5388 (47.3)	0.892 (0.859–0.926)	35.5	< 0.0001
Myocardial infarction	267	160 (59.9)	1.496 (1.171–1.911)	10.6	0.0011
Acute renal dysfunction	480	301 (62.7)	1.683 (1.399–2.026)	31.4	< 0.0001
Urinary tract infection	8984	5,182 (57.7)	1.378 (1.321–1.438)	220.4	< 0.0001
Postoperative cognitive dysfunction	3981	2,346 (58.9)	1.443 (1.354–1.538)	129.6	< 0.0001
Mortality during hospitalization	5082	3,471 (68.3)	2.190 (2.062–2.325)	709.5	< 0.0001

*P-values* of < 0.001 are considered significant by the χ2 test; CI means confidence interval

Table 4 shows the results of a multivariate logistic regression analysis to identify risk factors for mortality during hospitalization in patients with hip fractures. Several risk factors were identified as significantly associated with mortality: the odds ratio for being 90 years or older was 2.217 (95% CI: 2.088–2.354), p < 0.0001), and being male had an odds ratio of 2.567 (95% CI: 2.412–2.731, p < 0.0001). In addition, the odds ratio for elective surgery performed three or more days after admission was 1.191 (95% CI: 1.126–1.260, p < 0.0001), chronic renal dysfunction was 2.078 (95% CI: 1.889–2.286, p < 0.0001), dementia was 1.146 (95% CI: 1.077–1.219, p < 0.0001), and chronic lung disease was 2.006 (95% CI: 1.707–2.358, p < 0.0001).

**Table 4:** Multivariate logistic analysis for risk factors for mortality during hospitalization

Variable	Odds Ratio (95% CI)	χ2 statics	p-value
Super-elderly age	2.217 (2.088–2.354)	726.5	< 0.0001
Sex (Male)	2.567 (2.412–2.731)	780.3	< 0.0001
Surgery after 3 days of admission	1.191 (1.126–1.260)	37.1	< 0.0001
Hypertension	0.763 (0.720–0.810)	81.9	< 0.0001
Diabetes	1.133 (1.045–1.228)	9.1	0.0027
Cerebrovascular disease	1.134 (1.037–1.240)	7.4	0.0065
Chronic renal dysfunction	2.078 (1.889–2.286)	190.6	< 0.0001
Ischemic heart disease	1.043 (0.948–1.148)	0.8	0.38
Dementia	1.146 (1.077–1.219)	18.5	< 0.0001
Chronic lung disease	2.006 (1.707–2.358)	59.8	< 0.0001

P-values of < 0.001 are considered significant by the χ2 test; *CI* means confidence interval

**Fig. 2:**
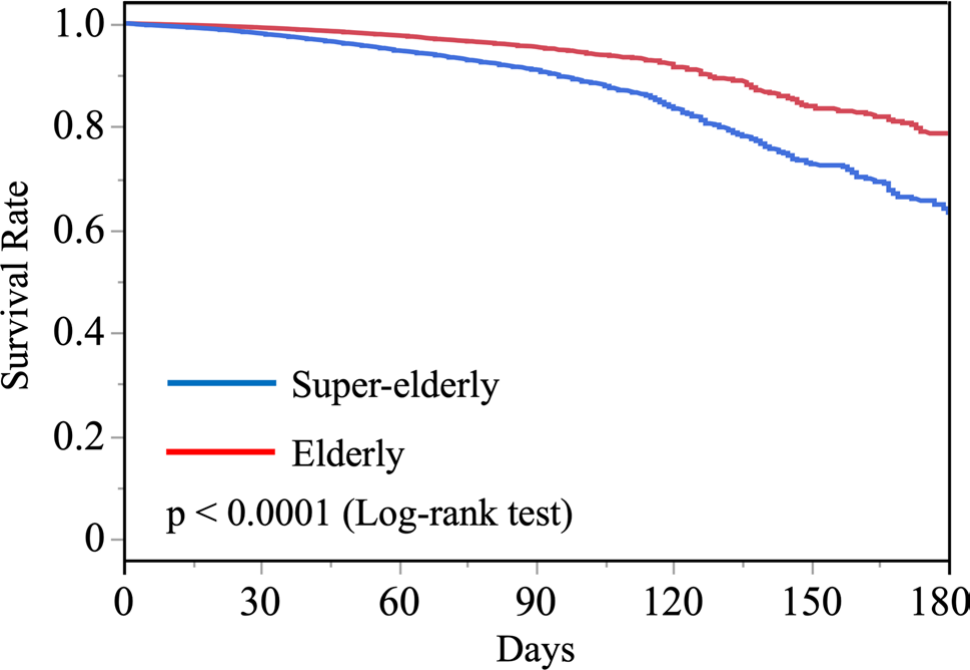
Survival rates of elderly and super-elderly groups. Results are expressed in the Kaplan–Meier curve. p < 0.0001 by log-rank test

Figure 2 shows the survival rates after surgery for hip fractures. The 30-day survival rate was 99.1% in the elderly patient group and 97.9% in the super-elderly patient group. Although the 30-day survival rate was satisfactory in both groups, the log-rank test showed that the survival rate of the super-elderly group was significantly lower than that of the elderly group throughout the entire period of continued hospitalization (p < 0.0001).

## Discussion

This study, the largest to date on hip fractures in the super-elderly population, used a comprehensive database of hip fractures in the elderly in Japan to analyze whether hip fractures in the super-elderly were associated with the incidence of pneumonia, pulmonary embolism, myocardial infarction, acute renal dysfunction, urinary tract infection, subsequent cognitive dysfunction, and in-hospital mortality. The results of this study showed that compared to hip fractures in the elderly aged 65 to 89, hip fractures in the super-elderly aged 90 and over were associated with an increased risk of pneumonia, urinary tract infection, acute renal dysfunction, subsequent cognitive dysfunction, and mortality during hospitalization, after adjusting for confounding factors. The odds ratios were 1.636 (95% CI 1.565–1.709), 1.683 (95% CI 1.399–2.026), 1.378 (95% CI 1.321–1.438), 1.378 (1.321–1.438), and 2.190 (95% CI 2.062–2.325), respectively. In addition, although 30-day survival was good at 99.1% for the elderly and 97.9% for the super-elderly, an association was found between being over 90 years of age and increased mortality during hospitalization. As hip fractures in the super-elderly are associated with a risk of serious subsequent complications and death, it was confirmed that prevention of falls and osteoporotic fractures, as well as early surgery after a fracture, are important.

There have been reports on the risk of mortality after a hip fracture, including advanced age, male sex, elective surgery after 48 h of injury, dementia, and chronic renal function [21–26]. This study also found that these variables were independently associated with the risk of mortality. Pneumonia-induced respiratory failure and myocardial infarction are reported to be the most common causes of death [15, 27]. In this study, the 30-day survival rate for the super-elderly group was 97.1%, which was a good result compared with previous overseas studies. Japan's medical system, which is making progress in dealing with an aging society, is thought to be becoming more adept at dealing with hip fractures in the super-elderly, such as perioperative management. However, this study did not examine the course of events after discharge from the hospital, and it did not examine the long-term prognosis, such as the more important one-year mortality rate, so it is necessary to clarify this in future cohort studies that also include data after discharge from the hospital.

The analysis of this study revealed details of hip fractures in Japanese super-elderly population. In the super-elderly group, the proportion of femoral neck fractures decreased (36.6%), while the proportion of femoral trochanteric fractures increased (61.2%) compared to the elderly group. The proportion of patients who underwent surgery within two days of admission was 51.1%, which is higher than the rate for the elderly group (47.1%). The implementation of antithrombotic treatments to prevent thrombosis and pulmonary embolism was 49.2% for both the super-elderly group and the elderly group, suggesting that the importance of preventing thrombosis is recognized within the Japanese healthcare system, even for the super-elderly population. However, since pneumonia, pulmonary embolism, and mortality rates during hospitalization are high in the super-elderly group aged 90 and over, it is hoped that in the future, hip fracture patients in this super-elderly group will receive even faster surgery and more thorough antithrombotic treatments to prevent embolism. There have been reports that the intervention of a multidisciplinary medical team before surgery was useful in reducing complications and shortening the length of hospital stay for elderly patients with hip fractures [28]. On the other hand, the use of bisphosphonates for the treatment of osteoporosis is low even among super-elderly patients, and the use of osteoanabolic agents and anti-RANKL antibodies, which are more effective in the treatment of osteoporosis than bisphosphonates [29, 30], is also low, suggesting that the treatment of osteoporosis is not yet sufficiently widespread. Because hip fractures in the super-elderly are associated with a high risk of serious complications, it is important to prevent them by thoroughly treating osteoporosis and promoting fracture liaison services [31].

There are several limitations to this large study, which will be discussed below. First, the study population included patients with hip fractures who were treated exclusively in acute care hospitals and reported in the DPC data system. This excludes patients admitted to non-DPC-reported beds, which represent 30% of all general hospital beds, or patients never treated in an acute hospital [16]. Secondly, the limitations of this study include the inability to validate the names of DPC diseases and the inability to assess the severity of symptoms in actual patients. Third, although conservative treatment may be chosen instead of surgery for super-elderly patients with hip fractures, this study only includes patients with surgical treatment, so the outcomes of conservative treatment in super-elderly patients are unexplored. Finally, another limitation of the study is that the risk of death in the long term after discharge from the hospital was not assessed; further large-scale studies based on real patient data are needed. In conclusion, this study, the largest on hip fractures in the super-elderly, analyzed data from Japan to examine the sequelae in those aged 90 and older. Compared with those aged 65–89 years, the super-elderly had a significantly higher risk of pneumonia, urinary tract infection, acute renal dysfunction, subsequent cognitive dysfunction, and in-hospital mortality. Despite high 30-day survival rates (99.1% for the elderly and 97.9% for the super-elderly), in-hospital mortality was higher for those over 90 years of age. As this study has revealed that the risk of sequelae of hip fractures and postoperative mortality is high in the super-elderly, it is necessary to re-recognize the importance of fall and fracture prevention, osteoporosis treatment, and early surgery in the super-elderly population.

## Supplementary Information

Below is the link to the electronic supplementary material.

Supplementary file1 (DOCX 24 KB)

## Abbreviations

CI: Confidence interval
DPC: Diagnosis procedure combination
PS: Propensity score
