# Supplementary material for: Nationwide database study of postoperative sequelae and in-hospital mortality in super-elderly hip fracture patients
Source: J Bone Miner Metab. 2024 Nov 7;43(2):141–8. doi: 10.1007/s00774-024-01564-w (PMC11993445; doi:10.1007/s00774-024-01564-w)
Supplement: Supplementary file 1 — Supplementary file1 (DOCX 24 KB) [file 774_2024_1564_MOESM1_ESM.docx]

Supplemental Table 1 Incidence of postoperative sequelae and in-hospital mortalities in each age group among elderly patients with hip fractures

|  | Total | Aged 65 to 79 | Aged 80 to 89 | Aged 90 and over |
| --- | --- | --- | --- | --- |
| Patients after PS matching | 259,906 | 39640 | 90313 | 129953 |
| Pneumonia | 8,684 | 749 (8.6) | 2,579 (29.7) | 5,356 (61.7) |
| Pulmonary embolism | 11,398 | 1,807 (15.9) | 4,203 (36.7) | 5,388 (47.4) |
| Myocardial infarction | 267 | 21 (7.9) | 86 (32.3) | 160 (59.9) |
| Acute renal dysfunction | 480 | 38 (8.0) | 141 (29.3) | 301 (62.7) |
| Urinary tract infection | 8,984 | 817 (9.1) | 2,985 (33.2) | 5,182 (57.7) |
| Postoperative cognitive dysfunction | 3,981 | 296 (7.4) | 1,339 (33.7) | 2,346 (58.9) |
| Mortality during hospitalization | 5,082 | 338 (6.7) | 1,263 (25.0) | 3,471 (68.3) |

PS means propensity score.
